# Beyond Early Excision and Grafting: A Perspective on Tissue-Preserving and Regenerative Topical Burn Wound Care

**DOI:** 10.3390/jcm15187033

**Published:** 2026-09-11

**Authors:** Hajime Matsumura, Miki Fujii

**Affiliations:** Department of Plastic and Reconstructive Surgery, Tokyo Medical University, Tokyo 160-0023, Japan

**Keywords:** burn wound, epithelialization, burn depth, hypertrophic scar, enzymatic debridement, biofilm, artificial dermis, dermal matrix, autologous skin cell suspension, cultured epidermal autograft

## Abstract

Whilst early debridement and autologous skin grafting remain the cornerstones of treatment for deep burns, there has been a shift towards a more selective and regenerative medicine-oriented approach in the topical management of burn wounds. The most critical issue is whether the wound can achieve epithelialization within approximately 2 to 3 weeks. This timeframe is of clinical significance due to the strong association between delayed epithelialization and hypertrophic scarring, which can result in contractures and functional impairment. However, given that the likelihood of epithelialization is influenced by factors such as the mechanism of injury, anatomical location and skin thickness at that site, age, and blood flow, its prediction remains incomplete and relies heavily on experience. The second challenge is to remove necrotic tissue while preserving as much healthy tissue as possible, thereby creating a wound bed with an adequately controlled microbial burden. Excessive excision should be avoided. The cytotoxic effects of several topical antimicrobial agents can inhibit the migration of keratinocytes, fibroblasts and other cells, as well as damaging the extracellular matrix. In order to address these issues, a range of methods are employed, including enzymatic debridement. The third challenge pertains to the reconstruction of the dermis in full-thickness burns, and the role of dermal and matrix-based materials is expanding. In addition to conventional artificial dermis, acellular fish skin matrices, synthetic biodegradable temporary matrices, and recombinant biomaterials are now being used. These materials can be regarded not solely as wound dressings, but also as instruments for preserving or re-establishing a biologically functional dermal matrix prior to epithelialization. Once these conditions are achieved, autologous skin cell suspension or cultured epidermal autografting may become appropriate options in selected wounds. This Perspective proposes a framework for precision-oriented local burn wound care structured around four sequential but iterative objectives.

## 1. Introduction: Changes in Treatment Strategies for Burn Wounds

Early surgical excision and skin grafting changed modern burn surgery [1]. Timely removal of nonviable tissue and definitive closure reduced the prolonged exposure of patients to an open necrotic wound and established active surgical management as a central principle of burn care. That principle remains valid. The question today is not whether early wound closure matters, but whether every wound that requires intervention should follow the same pathway of surgical excision followed by conventional autologous grafting. The aim of this framework is not to replace the established principles of timely excision and autologous grafting, which remain lifesaving and indispensable in appropriately selected deep burns, but to refine treatment selection when viable tissue can potentially be preserved or regenerative options can reduce unnecessary tissue loss or donor-site morbidity.

The therapeutic landscape of the burn wound has expanded. Selective bromelain-based enzymatic debridement can remove eschar while aiming to preserve viable dermal structures [2]. Objective perfusion imaging can assist assessment of healing potential [3,4]. Autologous skin cell suspension can reduce donor-skin requirements in selected burns [5]. A small case series reported early wound closure with favorable scar and cosmetic outcomes [6]. Cultured epidermal autografts can provide epithelial coverage when donor skin is severely limited [7,8]. Dermal substitutes can preserve, replace, or rebuild a dermal substrate before definitive epithelial closure [9,10,11,12,13]. These technologies create opportunities, but they also make treatment selection more complex.

This Perspective is deliberately restricted to topical/local burn wound management. It does not address fluid resuscitation, inhalation injury, critical-care management, systemic hypermetabolism, or other whole-patient aspects of severe burn care. Its focus is the biological and surgical sequence at the wound surface: how to determine whether a wound can heal acceptably on its own, how to remove what cannot survive, how to control microbial barriers without damaging healing tissue, how to preserve or reconstruct the dermis, and how to choose among spontaneous epithelialization, cell-based therapy, cultured epidermis, grafting, and combined approaches.

Our central argument is that the bottleneck in contemporary topical burn care is shifting from the availability of treatment options to the accuracy of treatment selection. More therapies are useful only if clinicians can identify the wound in which each therapy is biologically appropriate.

Importantly, the four-step framework proposed here should be regarded as a conceptual synthesis rather than a prospectively validated clinical algorithm. Each component is anchored in established clinical principles and published evidence—healing-potential assessment [3,4,14], selective debridement [2,15], dermal or matrix-based reconstruction [9,10,11,12,13,16,17], and cell-based or cultured epidermal closure [5,6,7,8,18]—but the integrated sequence itself has not yet been prospectively validated and requires further clinical evaluation.

## 2. Predict: Can This Wound Epithelialize Within Two to Three Weeks?

For partial-thickness burns, perhaps the most important early clinical question is not simply “How deep is this burn?” but “Can this wound epithelialize within approximately two to three weeks?” The distinction matters because burn-depth terminology is descriptive, whereas the decision faced by the clinician is prognostic. What matters to the patient is whether the wound will close in a time frame compatible with an acceptable cosmetic appearance and preservation of function without contracture.

The relationship between healing time and hypertrophic scarring is well established. In a prospective pediatric cohort, Cubison and colleagues demonstrated a progressive increase in hypertrophic scarring as time to healing lengthened, supporting the long-standing clinical emphasis on closure within approximately 21 days [14]. The precise threshold is not biologically absolute, and risk varies among patients and anatomical sites, but the two-to-three-week interval remains a clinically useful decision window. A wound that epithelializes rapidly is more likely to heal with limited scar burden; a wound that remains open longer is increasingly exposed to persistent inflammation and subsequent hypertrophic scarring, contracture, and functional impairment.

Predicting that outcome is difficult because burn injury is heterogeneous. The mechanism of injury—flame, scald, contact, chemical, or electrical—changes the pattern and duration of energy transfer. Skin thickness varies by anatomical site. Age influences dermal thickness and regenerative reserve. Perfusion, edema, systemic disease, and local vascular status modify tissue survival. The zone of stasis can recover or progress, so the biological depth of the wound is dynamic rather than fixed at the moment of first examination [19].

In practice, mixed-depth burns challenge any binary treatment rule. Different regions within the same injury may have experienced different durations of thermal exposure and may retain different amounts of viable dermis. Anatomical variation in dermal thickness and adnexal density can further influence epithelialization potential. Objective imaging can improve assessment, but it does not eliminate the need for experienced clinical integration. The wound should therefore be considered a spatially heterogeneous field rather than assigned a single depth category across the entire injured area.

Clinical examination therefore remains indispensable but imperfect. Monstrey and colleagues noted that clinical assessment of burn depth is accurate in only approximately 60–75% of cases even in experienced hands [4]. Laser Doppler imaging has the strongest established evidence among objective techniques for predicting healing potential and can improve decision-making in indeterminate-depth burns [3,4]. Other optical and perfusion-based approaches continue to develop, and artificial-intelligence models may ultimately integrate wound appearance, mechanism, location, patient characteristics, and temporal change. The desired endpoint, however, should remain clinically meaningful: not merely a more precise depth label, but a probability of timely epithelialization.

This shift—from burn-depth classification to prediction of healing potential—is fundamental to precision topical burn care. If a wound is highly likely to epithelialize within two to three weeks, unnecessary excision and donor-site injury should be avoided. If timely spontaneous closure is unlikely, intervention should occur before prolonged open-wound time converts diagnostic uncertainty into preventable scar morbidity.

The functional and cosmetic consequences of regional treatment selection must also be considered. Grafting only selected deeper areas while allowing adjacent areas to heal spontaneously may produce a conspicuous patch-like appearance, whereas excision of an entire anatomical or aesthetic unit may sacrifice tissue that could otherwise have healed. Experienced burn surgeons therefore integrate predicted healing time, anatomical site, expected scar distribution, and functional and cosmetic priorities when deciding whether heterogeneous regions should be treated separately or as a unit.

## 3. Prepare: Necrotic Tissue, Infection, Biofilm, and the Wound-Bed Paradox

Accurate prediction alone is insufficient. A wound with intrinsic regenerative potential may still fail if the local environment is hostile to healing. Necrotic tissue acts as a physical and biological barrier. Clinically significant infection can destroy residual viable tissue. Biofilm can sustain microbial persistence and chronic inflammation and may contribute to delayed healing [20,21,22]. These problems become particularly relevant when cell-based or tissue-engineered therapies are used, because transplanted cells and newly forming matrix require a recipient bed capable of supporting attachment, survival, migration, and proliferation.

Debridement is therefore not simply a prelude to grafting; it is a means of converting the wound from a necrotic, inflammatory environment into a biologically competent recipient bed. In partial-thickness burns, the ideal debridement removes irreversibly damaged tissue without unnecessarily sacrificing viable dermis. In deeper wounds, it prepares a clean, vascularized surface for dermal reconstruction or grafting.

Microbial control introduces a second problem. Topical antiseptics and antimicrobial dressings are indispensable in selected wounds, yet antimicrobial potency and tissue compatibility are not synonymous. Experimental studies have demonstrated cytotoxic effects of several topical antimicrobial agents on cultured human keratinocytes and fibroblasts [23,24]. The relevance of in vitro toxicity may vary depending on concentration, exposure time, formulation, exudate, protein binding and the clinical wound environment. Nevertheless, this principle is important when the therapeutic objective is regeneration rather than the simple suppression of microbial growth.

Examples illustrate why this distinction is clinically relevant. In vitro studies have shown marked cytotoxicity to human keratinocytes and fibroblasts with chlorhexidine- and povidone-iodine-containing antiseptic preparations at therapeutic concentrations [23]. In a separate experimental comparison that integrated antimicrobial efficacy with fibroblast cytotoxicity, octenidine and polyhexamethylene biguanide (PHMB) showed the most favorable biocompatibility indices among the agents tested [24]. These findings should not be interpreted as a fixed classification of “cytotoxic” versus “safe” agents, because tissue compatibility depends substantially on concentration, formulation, exposure time, protein binding, and the biological state of the wound.

Too little antimicrobial or antibiofilm activity permits infection, persistent microbial burden, and biofilm to impair healing. Too much cytotoxic exposure can damage the very keratinocytes, fibroblasts, extracellular matrix, and nascent tissue required for repair. The optimal endpoint is not sterility at any biological cost. It is microbial control compatible with host-tissue preservation. We therefore refer to this phenomenon as a “wound-bed paradox”.

For the purposes of this Perspective, the term “wound-bed paradox” is used as conceptual shorthand for this potential trade-off. It should be regarded as a hypothesis-generating concept rather than a validated biological construct, because direct clinical evidence defining the optimal balance between antimicrobial efficacy and preservation of host cells and matrix remains limited.

This balance is especially important when regenerative therapies are planned. A cultured epithelial sheet, sprayed autologous cells, or a newly vascularizing matrix should not be expected to overcome an inadequately debrided, infected, or biologically hostile wound bed. Conversely, repeated aggressive antiseptic exposure immediately before or after application of vulnerable cells may undermine the intended therapy. Future burn-wound protocols should therefore define not only which antimicrobial strategy is used, but also its timing relative to debridement, matrix integration, and cell delivery.

## 4. Preserve: Early Debridement Should Not Mean Maximal Excision

Whereas the above PREPARE step focuses on removing barriers to healing, this PRESERVE step focuses on minimizing the loss of viable regenerative tissue during that process. Early excision remains lifesaving and function-preserving when full-thickness tissue must be removed [1]. The emerging refinement is that “early” should not automatically imply “maximal.” Deep partial-thickness burns frequently contain a mosaic of nonviable and potentially recoverable dermis. Tangential excision is effective but necessarily depends on intraoperative interpretation of bleeding, color, texture, and tissue resistance. When the endpoint is uncertain, viable tissue may be removed together with eschar.

Bromelain-based enzymatic debridement (Nexobrid; MediWound Ltd., Yavne, Israel) has made the principle of selective tissue preservation clinically tangible. Bromelain-based enzymatic debridement preferentially removes thermally denatured eschar while aiming to preserve viable tissue. However, complete eschar removal may not always be achieved when the necrotic burden is extensive [25]. European consensus guidance and recent systematic-review evidence support its role in early eschar removal while emphasizing appropriate patient and wound selection [2,15].

Preserved dermis matters because it contains extracellular architecture, vascular networks, and adnexal epithelial reservoirs that can contribute to re-epithelialization. The more functional dermis that remains, the less tissue must subsequently be replaced. After selective debridement, the wound can be reassessed: is the residual dermis sufficient for spontaneous epithelialization, does it require regenerative assistance, should a dermal substrate be reconstructed, or is definitive autografting required? Autologous skin cell suspension after enzymatic debridement has also been reported to facilitate early epithelialization with favorable cosmetic outcomes in carefully selected small deep partial-thickness burns [6].

Preservation of viable dermis should not, however, be interpreted as an absolute objective. In selected functional or aesthetic units, particularly when a sheet autograft is planned, residual epithelial appendages beneath a thin graft may contribute to inclusion cysts, epithelial bridging, or persistent draining areas. In such circumstances, more complete excision may provide a more predictable recipient surface for sheet grafting. Conversely, when spontaneous epithelialization, autologous skin cell suspension, or meshed autografting is planned, selective preservation of viable dermis may remain advantageous. The practical principle is therefore to preserve tissue only when doing so is compatible with the intended closure strategy and the expected functional and cosmetic outcome.

This creates a two-stage diagnostic model. The first prediction is made before debridement from clinical history, examination, perfusion, and imaging. The second prediction is made after removal of necrotic tissue, when the residual wound bed can be directly assessed. The post-debridement wound is often more informative than the pre-debridement eschar. In this sense, selective debridement is both treatment and diagnostic clarification.

## 5. Rebuild: Dermal Matrices as Biological Wound-Bed Reconstruction in Full-Thickness Burns

Preservation is possible only when useful dermis remains. In full-thickness burns or after necessary excision of deep injury, the clinician faces a different problem: how to reconstruct the dermal component before or together with epithelial coverage. This is where artificial dermis and matrix-based materials assume a central role.

These products should not be conceptualized merely as temporary covers. A dermal matrix can provide three-dimensional architecture for cellular infiltration, neovascularization, extracellular-matrix deposition, and formation of a vascularized neodermis or dermal-like substrate [16]. Once integrated, that substrate can support subsequent split-thickness grafting and, in selected strategies, cultured or cell-based epithelial reconstruction. The therapeutic sequence is therefore not simply “cover the wound and graft later,” but “rebuild a biologically competent dermal bed, then restore the epidermal component.”

This distinction is relevant to scar quality and function. Thin split-thickness autografting directly onto a poorly reconstructed deep wound can close the surface, but it cannot reproduce the mechanical and biological functions of native dermis. Matrix-assisted reconstruction attempts to restore part of that missing compartment. The trade-off is time: many matrices require a period of vascular integration before definitive epithelial closure, and that interval must be weighed against infection risk, patient condition, anatomical site, and the need for rapid closure.

Experience with cultured epidermal autograft (CEA) illustrates why the dermal substrate matters. Histological work with CEA over bilayer artificial dermis demonstrated progressive dermal maturation but also highlighted the fragility of epithelial attachment when the dermal-epidermal interface is immature [9]. Clinical experience and systematic-review evidence support hybrid strategies in which CEA is combined with autologous skin grafting [7,10]. Thus, regenerative epidermal therapy cannot be considered independently of the quality of the tissue beneath it.

## 6. The Matrix Landscape Is Expanding

### 6.1. Conventional Artificial Dermis

Collagen-based bilayer dermal regeneration templates established the principle that a deep wound can be reconstructed in stages: first by creating a vascularized dermal substitute and then by applying an epidermal cover. Their role in burns is well established, although infection, integration time, cost, and the requirement for staged procedures remain important limitations [17]. The current significance of conventional artificial dermis is that it provides the conceptual foundation against which newer matrices should be judged.

### 6.2. Other Emerging Dermal Matrices

The following examples are not intended to be comprehensive, but rather to illustrate three distinct matrices.

For consistency, the term “dermal matrix” is used here as an umbrella term for scaffold-based materials intended to preserve, replace, or reconstruct a dermal or dermal-like substrate. Product-specific descriptions are retained only when differences in composition or biological behavior are clinically relevant.

Acellular fish-skin matrices preserve the native extracellular matrix components and provide a biologically active scaffold for wound bed reconstruction. Their use is expanding from complex wounds to burns, with emerging studies evaluating not only healing but also long-term scar outcomes [26].

NovoSorb BTM (PolyNovo Limited, Port Melbourne, Australia) is a fully synthetic biodegradable polyurethane matrix that supports vascularized dermal regeneration before skin grafting. Recent burn studies suggest comparable wound closure to Integra, with differences in integration time, graft take, and infection profiles, although comparative evidence remains limited [11,12].

Recombinant silk-elastin is an emerging Japanese wound-healing scaffold. A 2025 phase III study showed effective wound-bed preparation, and the Silk-Elastin Wound Healing Sheet was subsequently approved in Japan for partial- and full-thickness wounds, including intractable wounds [27,28].

For burn care, the important point is not to assume that regulatory approval for wounds establishes a burn-specific standard of care. Burn-specific indications, timing, infection management, and comparative effectiveness still require definition. Its conceptual relevance, however, is substantial: silk-elastin is a recombinant biomaterial intended to influence the wound environment and tissue repair, illustrating the movement from passive coverage toward engineered regenerative matrices.

## 7. Regenerate or Replace: A Continuum Rather than Competing Technologies

Once healing potential has been predicted, necrotic and microbial barriers addressed, and the dermal substrate preserved or reconstructed, the final question is how much additional tissue must be supplied. The answer should be individualized according to the residual regenerative capacity of the wound and the time available for safe closure.

At one end of the continuum is spontaneous epithelialization. If sufficient viable dermis and epithelial reservoirs remain and closure is expected within an acceptable time frame, preservation may be the most biologically economical treatment. The objective is not therapeutic minimalism for its own sake, but avoidance of unnecessary donor-site injury.

Autologous skin cell suspension provides an intermediate strategy. RECELL (Avita Medical, Valencia, CA, USA) prepares a point-of-care suspension of autologous skin cells from a small donor sample. A small donor sample can be processed to treat a recipient area up to approximately 80 times the donor-site area. In a prospective randomized study of mixed-depth burns, RECELL combined with more widely meshed autograft reduced donor-skin requirements while achieving wound closure outcomes comparable to standard grafting [5]. A recent meta-analysis of randomized studies also found a modest reduction in time to re-epithelialization with autologous skin cell suspension, although no significant differences were observed in several secondary outcomes [18]. Its value therefore lies not in replacing grafting in every wound, but in changing the ratio between donor skin harvested and recipient area treated. Furthermore, in cases of deep partial-thickness burns where the possibility of spontaneous epithelialization is unclear, using this product alone—without mesh grafting—may facilitate early epithelialization in carefully selected small deep partial-thickness burns [6].

CEA occupies another position on the continuum. CEA has been used extensively in Japan for severe burns, particularly when donor sites are limited. Six-year multicenter surveillance documented its clinical use in extensive burns [7], and more recent registry-based propensity-score analysis reported an association between CEA use and improved survival in selected extensive-burn patients [8]. Although this Perspective is focused on the local wound, these data underscore the potential importance of expanding epithelial coverage when donor skin is scarce. Allogeneic skin grafts also remain valuable as temporary biological coverage in regions with established skin-bank systems, particularly in extensive burns when autologous donor skin is temporarily insufficient. However, availability depends heavily on local tissue-bank infrastructure, donor supply, and regulatory systems, which remain limited in many parts of the world. In addition, although modern donor screening and tissue-processing standards substantially reduce infectious risk, transmission of recognized or emerging pathogens cannot be completely excluded. These practical and safety constraints may limit the universal applicability of allogeneic skin as a broadly generalizable long-term strategy [29,30].

Conventional split-thickness autografting remains indispensable. A precision framework should not create a hierarchy in which newer technology is assumed to be superior. Rather, it should identify the least destructive strategy that can reliably achieve durable closure within a biologically appropriate time frame. In many wounds, that strategy will still be grafting. In others, it may be spontaneous healing, cell-based therapy, dermal reconstruction followed by grafting, CEA combined with meshed autograft, or another staged combination.

## 8. A Four-Step Framework for Precision Topical Burn Wound Care

**PREDICT:** Estimate the probability of epithelialization within approximately two to three weeks. Integrate injury mechanism, anatomical site and skin thickness, age, perfusion, wound evolution, clinical examination, and objective imaging. The endpoint is healing potential, not depth classification alone.

**PREPARE:** Remove necrotic tissue and establish microbial control. Address infection and, where clinically suspected, biofilm, but avoid unnecessary cytotoxic exposure that may damage viable host cells and matrix. The target is a regeneration-compatible wound bed.

**PRESERVE OR REBUILD:** Preserve native viable dermis whenever possible. If useful dermis has been lost, consider whether a dermal or matrix-based material can create a vascularized substrate suitable for subsequent epithelial reconstruction.

**REGENERATE OR REPLACE:** After reassessment, choose spontaneous epithelialization, autologous cell suspension, cultured epidermis, split-thickness grafting, or a combined/staged strategy. Replace only the tissue that cannot be expected to regenerate in an acceptable time frame.

**Figure 1:**
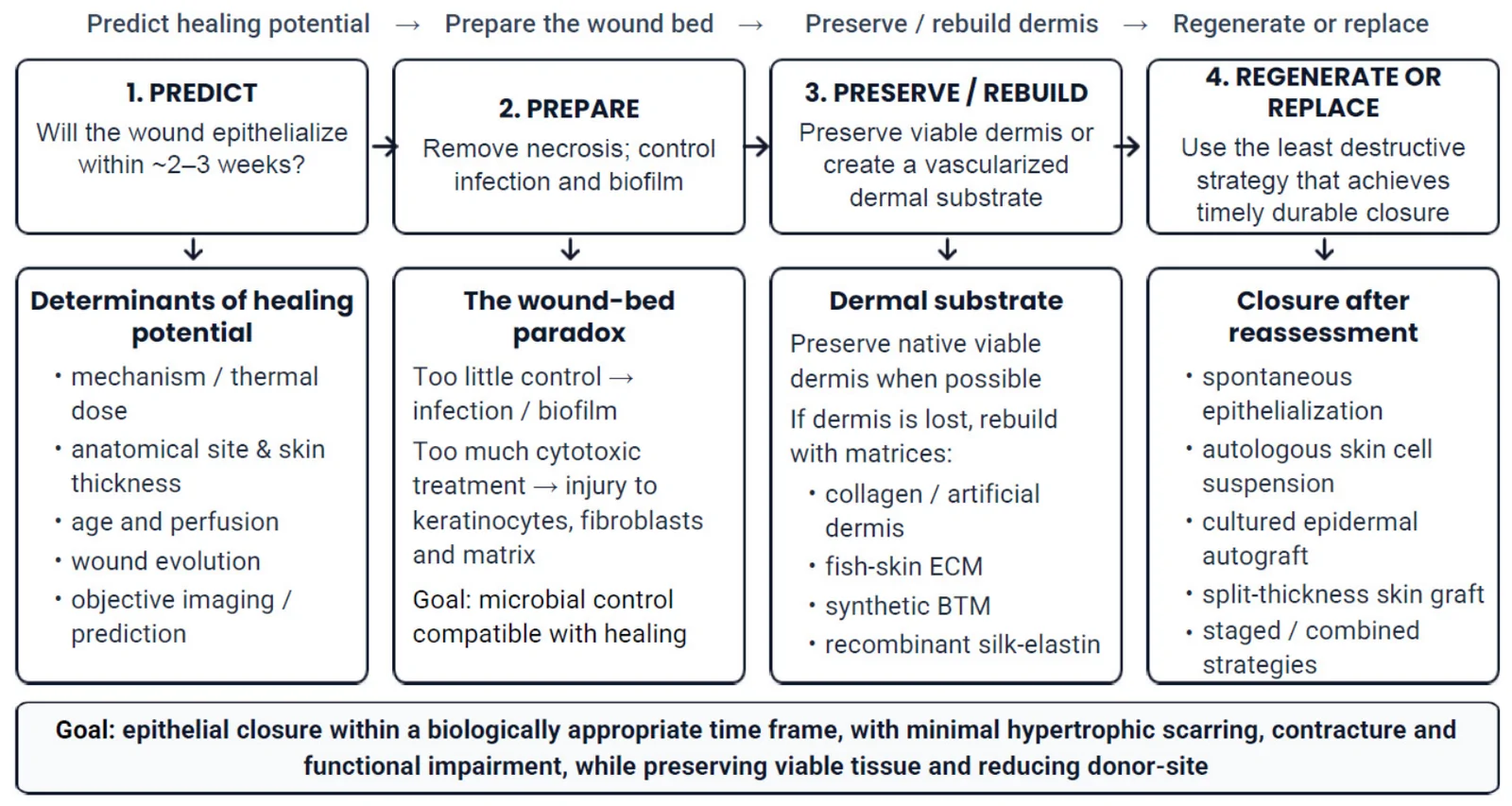
Conceptual framework for precision-oriented burn wound care. The central decision is whether timely epithelialization is likely. If intervention is required, the wound is prepared by debridement and microbial control, viable dermis is preserved or a dermal substrate is rebuilt, and the least destructive reliable closure strategy is selected after reassessment.

This framework emphasizes that each step changes the information available for the next. Initial prediction determines whether intervention is needed. Debridement reveals the residual tissue. Wound-bed preparation determines whether regenerative therapy is biologically plausible. Matrix integration may transform a deep defect into a graftable or cell-compatible recipient bed. Treatment selection should therefore be iterative rather than fixed at the first examination (Figure 1).

Two evidence-informed clinical scenarios illustrate how the sequence may be applied, without implying superiority over standard care. First, in a small mixed-depth deep partial-thickness burn of the hand or foot, initial assessment may leave uncertainty regarding spontaneous healing. Selective enzymatic debridement can expose the residual wound bed and permit reassessment; in carefully selected wounds, autologous skin cell suspension may support early epithelialization, whereas grafting remains appropriate if timely closure is unlikely [6]. If sufficient viable dermis and epithelial reservoirs remain after debridement, the strategy may remain within the PRESERVE pathway and allow spontaneous epithelialization; when healing potential is borderline but a biologically competent dermal bed remains, treatment may transition toward REGENERATE with autologous skin cell suspension. If timely epithelialization remains unlikely, the strategy shifts toward REPLACE with autologous skin grafting. Second, in a full-thickness burn in which useful dermis has been lost, complete excision may be followed by placement of a dermal matrix to establish a vascularized substrate before split-thickness autografting. In selected extensive burns in which donor skin is severely limited, cultured epidermal autograft may be considered as an adjunctive or staged epithelial coverage strategy [7,9,10,11,12,13]. These scenarios are illustrative applications of the framework rather than comparative evidence that the framework itself outperforms conventional treatment.

## 9. Future Directions

The next advance in topical burn care may not be a single new dressing, matrix, imaging device, or cell therapy. It may be the ability to connect diagnosis, wound-bed biology, dermal reconstruction, and epithelial closure into a reproducible treatment-selection system.

First, healing prediction must improve. The clinically useful output of future imaging and artificial-intelligence systems should be an individualized probability of epithelialization within a defined time window, ideally with uncertainty estimates and adjustment for anatomical site and patient factors. A binary label such as superficial or deep is less useful than a calibrated prediction that can be linked to treatment thresholds.

Second, the biology of the wound bed should become measurable. Current decisions about infection, biofilm, inflammatory burden, and readiness for regenerative therapy are often based on indirect clinical signs. Point-of-care methods that characterize microbial burden, perfusion, protease activity, inflammation, or other features of the wound microenvironment could help determine when a wound is ready for cells, matrices, or grafts.

Third, matrices should be evaluated according to function rather than category. Important outcomes include integration time, resistance or susceptibility to infection, ability to support vascularization, compatibility with cells and grafts, donor-site reduction, scar quality, elasticity, contracture, and long-term function. Head-to-head comparative studies remain limited, and recent meta-analytic evidence for BTM versus Integra illustrates both the promise and the low certainty of current comparative data [12].

Finally, regenerative therapies should be studied as components of sequences rather than isolated products. The effect of a cell therapy may depend on how the wound was debrided, which antimicrobial agents were used, whether dermis was preserved, whether a matrix was placed, and when cells were delivered. The clinically relevant intervention is often the entire pathway, not a single device.

The proposed framework also has important limitations. Burn wounds are heterogeneous across and within patients, and treatment selection must account for mechanism, anatomical site, wound depth, local dermal and adnexal anatomy, patient factors, functional and cosmetic priorities, and surgeon experience. The four steps are therefore intended to organize major decision domains rather than to encompass every nuance of burn surgery. Moreover, the framework has not yet been prospectively tested as an integrated algorithm, and future studies should evaluate whether its use improves decision consistency, donor-site burden, healing time, scar quality, function, or other clinically meaningful outcomes.

## 10. Conclusions

Early excision and grafting remain essential principles of burn surgery, but modern topical burn wound care can be more selective. The first challenge is to predict whether a partial-thickness wound can epithelialize within approximately two to three weeks, because timely closure is closely linked to scar and functional outcome. The second is to create a wound bed that is free of irreversibly damaged tissue and adequately controlled microbiologically without destroying its regenerative capacity. The third is to preserve viable dermis or, when dermis is lost, to rebuild a vascularized dermal substrate using appropriate matrix technologies. These considerations should then inform how much additional epithelial tissue, if any, needs to be supplied.

*Predict what can heal. Prepare the wound for healing. Preserve or rebuild the dermis. Regenerate what can regenerate, and replace only what cannot.*

## Data Availability

No new data were created or analyzed in this study. Data sharing is not applicable to this article.
